# CD146 Promoter Polymorphism (rs3923594) Is Associated with Recurrence of Clear Cell Renal Cell Carcinoma in Chinese Population

**DOI:** 10.1155/2017/2543059

**Published:** 2017-05-24

**Authors:** Gang Feng, Hou-Bao Huang, Xiao-Bing Ye, Peng Zhang, Jian-Jun Huang, Li-Zhu Huang, Long Cheng, Chun Pu, Guorong Li

**Affiliations:** ^1^Clinical Laboratory, The First Affiliated Hospital of Wannan Medical College, Wuhu, Anhui 241001, China; ^2^Department of Urology, The First Affiliated Hospital of Wannan Medical College, Wuhu, Anhui 241001, China; ^3^Department of Oncology, The First Affiliated Hospital of Wannan Medical College, Wuhu, Anhui 241001, China; ^4^Department of Urology, North Hospital, CHU of Saint-Etienne, 42055 Saint-Etienne, France

## Abstract

**Introduction:**

CD146 is a membrane signal receptor in tumor-induced angiogenesis. However, limited studies have focused on the CD146 promoter polymorphisms in clear cell renal cell carcinoma (ccRCC).

**Purpose:**

The purpose of this study was to investigate the association between polymorphisms located in the promoter region of the CD146 gene and characteristics of ccRCC in Chinese population. The association between the CD146 promoter polymorphisms and CD146 expression was also investigated in ccRCC.

**Materials and Methods:**

A total of 600 samples including 300 ccRCC patients and 300 healthy controls were collected for analysis of the CD146 promoter polymorphisms by direct sequence. The CD146 expressions were measured by qRT-PCR.

**Results:**

We had not found any significant differences in genotypic and allelic frequencies of CD146 promoter polymorphisms between ccRCC patients and controls. The rs3923594 was associated with stage and metastasis (300 cases) and recurrence (263 cases) of ccRCC in Chinese population. A significant association was also observed between the rs3923594 and CD146 expression (227 cases) in ccRCC.

**Conclusions:**

CD146 promoter polymorphisms were not associated with the risk of ccRCC in Chinese population. The rs3923594 was an independent predictor of recurrence in Chinese patients with localized ccRCC.

## 1. Introduction

Renal cell carcinoma (RCC) is a common solid renal malignancy in adults worldwide [1]. In China, the incidence of RCC ranks second among malignant tumors of the urinary system, with an average increase rate at 7.6% per year [2]. The clear cell RCC (ccRCC) accounts for 70% to 85% of all kidney cancers and is the most aggressive subtype with the highest rate of metastasis and mortality [3].

Most of the patients with ccRCC have presented with a localized disease at the time of diagnosis. The goal of surgery is to cure these patients. Unfortunately, up to 40% of all patients will present with recurrent disease after surgery. The recurrence of ccRCC usually occurs within 3 years after surgery [4]. Thus, there is a need to screen patients who are at risk of recurrence after surgery.

CD146, also known as MCAM or MUC18, is a membrane calcium-independent glycoprotein adhesion molecule, which was identified as a tumor angiogenesis marker [5]. Previous studies had confirmed that CD146 presented in most blood vessels and might involve metastasis via the vascular system in cervical and endometrial cancer [6, 7]. Our previous study had also reported that CD146 was overexpressed in ccRCC and associated with metastasis or recurrence of ccRCC [8].

Many studies have explored that polymorphisms located in the promoter region of a gene could alter gene expression by affecting the transcriptional rate [9]. Thus, we speculate that the polymorphisms located in the promoter region of the CD146 gene might be associated with the abnormal CD146 gene expression. The present study aimed to investigate the association between polymorphisms located in the promoter region of the CD146 gene and characteristics of ccRCC in Chinese population. The association between the CD146 promoter polymorphisms and CD146 gene expression was also investigated in ccRCC.

## 2. Materials and Methods

### 2.1. Patients and Healthy Controls

Peripheral blood samples were obtained from 300 sporadic ccRCC patients and 300 controls between February 2012 and May 2016. All the patients had histopathologically confirmed incident ccRCC and had undergone radical or partial nephrectomy. Tumor stage was assigned according to the 2010 American Joint Committee on Cancer (AJCC) TNM (tumor-node-metastasis) classification. Tumor nuclear grading was based on the criteria of Fuhrman by a single pathologist. Patients with an M classification of 0 or an N classification of 0 were considered to have localized disease (M0N0). The presence or absence of necrosis and the extent of necrosis were evaluated for all specimens. Estimation of the percentage of tumor necrosis was based on the imageological description of the tumor. The extent of necrosis was graded according to the following scale: 0, no necrosis; 1, <25% necrosis; 2, 25–50% necrosis; 3, 51–75% necrosis; and 4, 76–100% necrosis [10].

None of the patients had ever received immunotherapy or targeted therapies before surgery. A total of 300 controls were recruited from healthy volunteers who went for regular physical check-up at our hospital and were frequency-matched to the cases on sex and age. All study subjects were all of ethnic Chinese origin and provided with written consent. The study protocol was approved by the Institutional Ethical Review Board of Wannan Medical College.

### 2.2. Follow-Up

The follow-up program after operation consisted of a clinical examination, laboratory tests, and a pulmonary X-ray at 3 months, then every 6 months during the first 3 years, and thereafter examination yearly. Abdominal computed tomography was performed at 6 and 24 months after nephrectomy and additional imaging used whenever indicated. All patients had adequate information for follow-up. The maximum follow-up time was 54 months (last follow-up in August 2016) and the median follow-up time was 24.5 months. The recurrence-free survival (RFS) time was calculated from the date of operation until a patient's known recurrence or until the date of the last follow-up visit.

### 2.3. DNA Isolation and Genotyping

Total genomic DNA was isolated from venous blood using the QIAamp DNA Mini Kit (Qiagen S.A.) according to the manufacturer's protocol. Four fragments located in the promoter region of the CD146 gene were amplified, respectively. The sequences and positions of primers are shown in Table 1. The reactions were performed in a total volume of 25 *μ*l containing 1 *μ*g genomic DNA, 5 *μ*M of each primer, and 12.5 *μ*l 2× PCR Master Mix (Thermo Scientific; Pittsburgh, PA, USA). The cycling parameters for PCR were as follows: denaturation at 94°C for 4 min, followed by 35 cycles of denaturation at 94°C for 40 s, annealing at 59°C for 30 s, and extension at 72°C for 60 s. Specificity of PCR product was confirmed by 5% polyacrylamide gel electrophoresis, and products were sequenced by an ABI3730xl DNA Analyzer.

### 2.4. CD146 Expression

All tumor samples were obtained immediately after the operation and put into a liquid nitrogen freezer for storage. The total RNA was isolated (RNeasy Mini Kit, Qiagen S.A.) and transcribed to cDNA (High-Capacity RNA-to-cDNA Kit; Life Technologies). Amplification and detection of CD146 mRNA were performed as previously described [8]. CD146 gene expression was defined as a ratio of CD146 mRNA to beta-actin mRNA and then multiplied by 1000 for easier tabulation.

### 2.5. Statistical Analysis

All allele frequencies of polymorphism were tested against departure from Hardy-Weinberg equilibrium (HWE) before the analysis. The demographic characteristics, selected variables, and frequencies of genotypes in patients and controls were compared by Student's *t*-test for continuous variables and chi-square test or Fisher's exact test for categorical variables. The associations between the polymorphisms and ccRCC risk were estimated by computing the odds ratios (ORs) and 95% confidence intervals (CIs) from unconditional logistic regression analysis with the adjustment for possible confounders. Univariate or multivariate Cox regression analysis was done to determine predictive factors of recurrence of localized ccRCC by estimating the hazard ratios (HRs) and their 95% CIs. The recurrence-free survival curves were calculated by the Kaplan-Meier method and compared by the log-rank test. All statistical analyses were performed using SPSS 13.0 software (SPSS Inc.). *P* < 0.05 in a two-side test was considered statistically significant.

## 3. Results

### 3.1. Characteristics of Patients with ccRCC and Controls

The distributions of selected characteristics of the 300 ccRCC patients and 300 controls are shown in Table 2. There were no significant differences between patients and controls according to age (*P* = 0.994) and sex (*P* = 0.248).

### 3.2. Genotype Distribution of CD146 Promoter Polymorphisms in ccRCC Patients and Controls

We had detected six single nucleotide polymorphisms (SNPs) of CD146 promoter (Table 3). The genotype frequencies of all SNPs were conformed to Hardy-Weinberg equilibrium (all *P* > 0.05). No significant differences in genotype and allele frequencies were observed between patients with ccRCC and controls (Table 3).

### 3.3. Associations between CD146 Promoter Polymorphisms and Clinicopathological Characteristics of ccRCC

There were not any significant associations between five SNPs and clinicopathological characteristics of ccRCC in this study. Significant associations were found between rs3923594 and stage (*P* = 0.030), metastasis in lymph node (*P* = 0.007), and distant metastasis (*P* = 0.005) (Table 4).

### 3.4. Associations between CD146 Promoter Polymorphisms and Recurrence of Localized ccRCC

**Figure 1:**
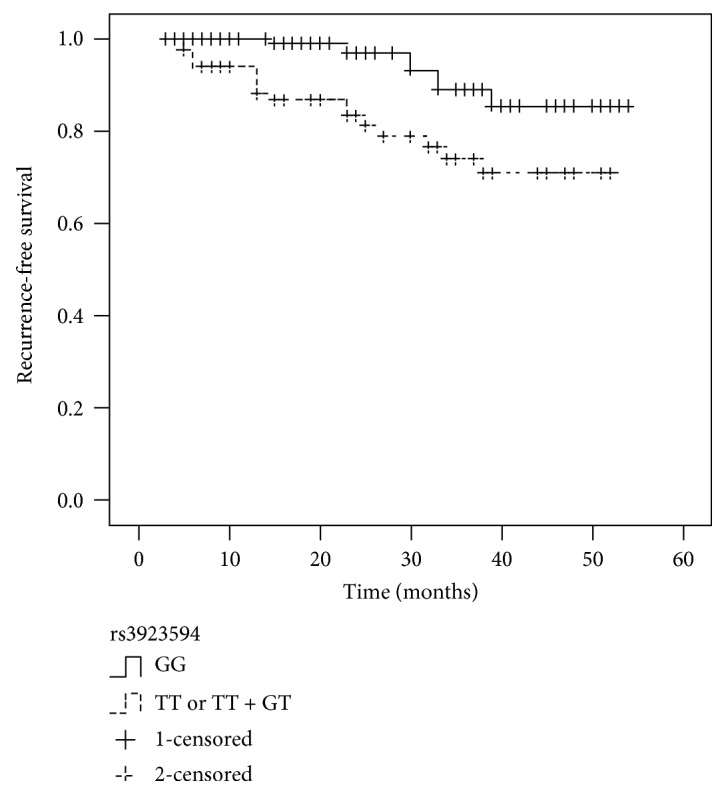
Kaplan-Meier survival curves illustrate recurrence-free survival according to the genotype of rs3923594. Patients with the TT or GT + TT had a significantly higher recurrence rate than those with the GG (*P* = 0.023).

None of significant associations were found between five SNPs and recurrence of localized ccRCC. As shown in Table 5, the rs3923594 was significantly associated with recurrence of localized ccRCC (HR, 2.788; 95% CI, 1.186–6.586; *P* = 0.021) by the univariate analysis. A multivariate Cox proportional hazard model indicated that tumor size, stage, grade, presence of necrosis, and rs3923594 (all *P* < 0.05) were independent predictors of recurrence of localized ccRCC. Kaplan-Meier analyses were applied to compare RFS in patients with different genotypes of rs3923594 (TT or GT + TT, GG). The patients (M0N0) with the TT or GT + TT genotype demonstrated a significantly lower RFS (*P* = 0.023) compared with those with the GG genotype (Figure 1).

### 3.5. Associations between CD146 Promoter Polymorphisms and CD146 Gene Expression at mRNA Level

**Figure 2:**
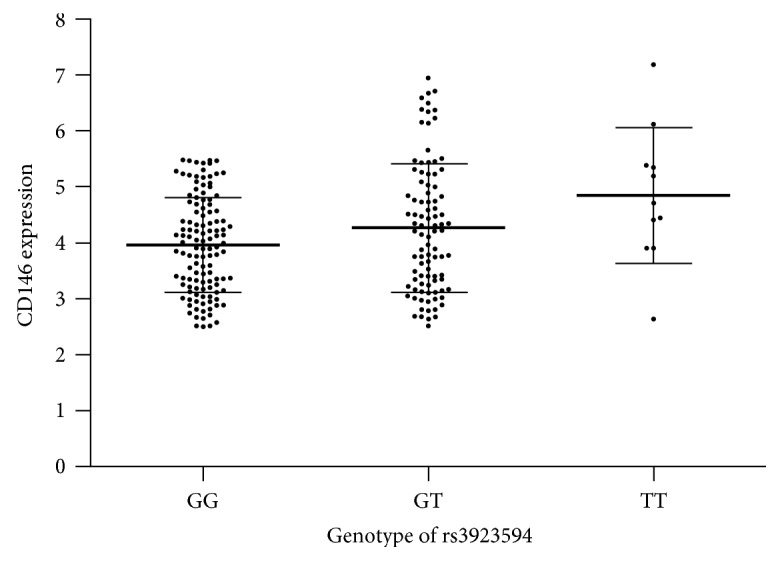
CD146 expression at mRNA levels in groups with different rs3923594 genotypes.

None of significant associations were found between five SNPs with CD146 gene expression, whereas rs3923594 was associated with CD146 gene expression at mRNA level in 227 specimens. The mean of CD146 gene expression in patients with genotype TT of rs3923594 (4.84 ± 1.22) was significantly higher than that in those with genotype GG (3.96 ± 0.85, *P* = 0.014). The mean of CD146 gene expression in patients with genotype GT of rs3923594 (4.26 ± 1.14) was also higher than that in those with genotype GG, but the difference did not reach statistical significance (*P* = 0.119) (Figure 2).

## 4. Discussion

Continuous and frequent radiological examinations, such as CT, MRI, or bone scintigraphy, have served an important role in monitoring recurrence of localized ccRCC during follow-up [11]. Compared with those of more expensive imaging methods, analyses of tumor biomarkers have no risk of radiation exposure and are easily available and cost-effective.

Currently, many studies have reported that polymorphism of NFKB1 [12], PTPRD [13], CASP8 [14], GSTM3 [15], and BTLA [16] is related to the risk of RCC. Some researchers have further presented the associations between survival of RCC and polymorphism of IL-4 [17], VEGF [18], CYP3A5, and ABCB1 [19].

In this study, we had detected six SNPs located in the promoter region of the CD146 gene. Firstly, we had investigated the associations between SNPs and the risk of ccRCC in Chinese population. The genotype distributions of all SNPs were in HW in the control group, which indicated no population stratification. Between ccRCC patients and controls, we had not found any significant differences in the distribution of genotypes of all SNPs. Therefore, there was no evidence of the association between these CD146 promoter SNPs and the risk of ccRCC in Chinese population.

We had investigated the associations between CD146 promoter polymorphisms and clinicopathological characteristics of ccRCC in Chinese population. However, the rs543070476, new 1, new 2, new 3, and new 4 were not associated with clinicopathological characteristics of ccRCC. The significant associations were found between rs3923594 and stage, lymph node metastasis, and distant metastasis. These results suggested that the rs3923594 was associated with tumor stage and metastasis of ccRCC in Chinese population. None of significant association was found between the rs3923594 and grade in this study. Although only 17 patients have grade 3 or 4 in our study, this result needs further validation.

We also investigated the associations between rs3923594 and recurrence of localized ccRCC in Chinese population. Univariate analysis demonstrated that the rs3923594 was significantly associated with recurrence of localized ccRCC. Furthermore, multivariate analysis reconfirmed the independence of rs3923594 in predicting recurrence, just like tumor size, stage, grade, and presence of necrosis. Thus, rs3923594 may serve as a valuable predictor of recurrence in patients with localized ccRCC.

We had found the association between the rs3923594 and CD146 gene expression at mRNA level in patients with ccRCC. However, the specimens of small tumors (diameter ≤ 2 cm, 73 cases) had not been collected in this study, which might reduce the reliability of this result. We had also found that the mean of CD146 gene expression at mRNA level in patients with genotype GG of rs3923594 was lowest. However, the higher CD146 gene expressions were also found in small number of these patients. We consider that the promoter polymorphism may be just one of the factors that affect CD146 gene expression. The exact mechanism remains to be elucidated in the future.

When interpreting our results, some limitations need to be considered. Firstly, some risk factors of ccRCC, such as hypertension, obesity, and smoking, were not collected in our analyses. Secondly, because of the small sample size and the short follow-up, statistical significance should be interpreted with caution. Thirdly, CD146 gene expression had been measured in only partial patients. Further validation in a larger population and functional characterizations are needed in our future studies.

## 5. Conclusion

In summary, CD146 promoter polymorphisms were not associated with the risk of ccRCC in Chinese population. The rs3923594 was an independent predictor of recurrence in Chinese patients with localized ccRCC.

## Figures and Tables

**Table 1:** The sequences of primers, position of amplification, and length of product.

		Sequence (5′ → 3′)	Start	Stop	Fragment
Primer 1	Forward	CCCGAGACACCTGTCACTAC	+17	−242	259 bp
	Reverse	GAGGGCGAGAGCCAAGTGAG			
Primer 2	Forward	TGGTGCAATCGTTCTGGGAA	−216	−494	269 bp
	Reverse	GGTAGTGACAGGTGTCTCGG			
Primer 3	Forward	GTTTGAAGGGACAGCCCAGA	−469	−628	160 bp
	Reverse	GCTTCCCAGAACGATTGCAC			
Primer 4	Forward	ACTGAGTTCCAGGGTAGGCT	−613	−775	162 bp
	Reverse	GCTGTCCCTTCAAACGCAAG			

Transcription start site is defined as +1.

**Table 2:** Distribution of selected variables between the ccRCC patients and controls.

Variables	Patients	Controls	*P* value
Age (mean ± SD), year	58.2 ± 11.6	58.0 ± 11.1	0.994
≤57	136	142	0.623
>57	164	158	
Sex			0.248
Male	178	164	
Female	122	136	
Tumor size (diameter)			
≤2 cm	73		
>2 cm	227		
Tumor stage			
T1 + T2	259		
T3 + T4	41		
Tumor grade			
1 + 2	283		
3 + 4	17		
Extent of necrosis			
0	186		
1	67		
2	28		
3	15		
4	4		
Lymph node metastasis			
Negative	271		
Positive	29		
Distant metastasis			
Negative	270		
Positive	30		
Recurrence			
Negative	242		
Positive	21		

**Table 3:** Genotype and allele frequencies of CD146 promoter polymorphisms among the ccRCC patients and controls.

Genotype	Patients	Controls	*P* ^a^ value	OR^a^ (95% CI)
rs3923594				
GG	194	216	0.095	1.000 (reference)
GT	95	75	0.064	0.677 (0.571–0.992)
TT	11	9	0.463	0.713 (0.289–1.760)
G allele	483	507	0.068	
T allele	117	93		
rs543070476				
GG	236	249	0.393	1.000 (reference)
GA	55	44	0.206	0.753 (0.485–1.169)
AA	9	7	0.548	0.735 (0.268–2.011)
G allele	527	542	0.165	
A allele	73	58		
New 1				
CC	256	269	0.474	1.000 (reference)
CG	44	31	0.108	0.633 (0.439–1.119)
GG	0	0		
C allele	556	569	0.125	
G allele	44	31		
New 2				
AA	246	251	0.442	1.000 (reference)
AC	54	49	0.220	0.703 (0.336–1.155)
CC	0	0		
A allele	546	551	0.606	
C allele	54	49		
New 3				
CC	229	238	0.647	1.000 (reference)
CA	71	62	0.851	0.945 (0.640–1.285)
AA	0	0		
C allele	529	538	0.408	
A allele	71	62		
New 4				
TT	244	259	0.403	1.000 (reference)
TG	53	40	0.131	0.769 (0.517–1.098)
GG	3	1	0.060	0.698 (0.554–0.991)
T allele	541	558	0.077	
G allele	59	42		

^a^Adjusted for age and sex in logistic regression model.

**Table 4:** Associations of the rs3923594 with clinicopathologic variables of ccRCC.

	Genotype		
	GG	GT	TT
Stage			
T1 + T2	172	80	7
T3 + T4	22	15	4
OR^a^ (95% CI)	1.000 (reference)	1.505 (0.732–3.093)	4.287 (1.152–15.952)
*P* value		0.266	0.030
Grade			
1 + 2	183	91	9
3 + 4	11	4	2
OR^a^ (95% CI)	1.000 (reference)	0.735 (0.218–2.472)	3.150 (0.570–17.406)
*P* value		0.618	0.188
Metastasis in lymph node			
Negative	179	85	7
Positive	15	10	4
OR^a^ (95% CI)	1.000 (reference)	1.497 (0.639–3.508)	6.414 (1.668–24.661)
*P* value		0.353	0.007
Distant metastasis			
Negative	184	80	6
Positive	10	15	5
OR^a^ (95% CI)	1.000 (reference)	1.761 (0.828–4.125)	6.982 (1.788–29.654)
*P* value		0.114	0.005

^a^Adjusted for age and sex in logistic regression model.

**Table 5:** Univariate and multivariate Cox regression analysis in patients with localized ccRCC.

Variables	RFS					
	Univariate			Multivariate		
	HR	95% CI	*P* value	HR	95% CI	*P* value
Age (≤57 versus >57)	1.290	0.601–3.684	0.372			
Sex (male versus female)	1.481	0.714–3.576	0.382			
Tumor size (≤2 versus >2 cm)	2.109	1.240–4.843	0.006	1.853	1.008–3.451	0.023
Stage (T1 + T2 versus T3 + T4)	4.828	2.754–8.266	<0.001	2.963	1.438–5.157	0.005
Grade (1 + 2 versus 3 + 4)	3.996	2.212–7.249	<0.001	2.852	1.339–5.274	0.003
Presence of necrosis (yes versus no)	2.548	1.339–5.982	0.012	1.985	1.021–3.682	0.019
rs3923594 (GG versus GT + TT)	2.788	1.186–6.586	0.021	2.116	1.053–4.107	0.034
